# Upper Bound on the Joint Entropy of Correlated Sources Encoded by Good Lattices

**DOI:** 10.3390/e21100957

**Published:** 2019-09-29

**Authors:** Christian Chapman, Daniel W. Bliss

**Affiliations:** School of Electrical, Computer and Energy Engineering, Arizona State University, Tempe, AZ 85281, USA; d.w.bliss@asu.edu

**Keywords:** lattice codes, network information theory, distributed source coding, compressed sensing

## Abstract

Lattices provide useful structure for distributed coding of correlated sources. A common lattice encoder construction is to first round an observed sequence to a ‘fine’ lattice with dither, then produce the result’s modulo to a ‘coarse’ lattice as the encoding. However, such encodings may be jointly-dependent. A class of upper bounds is established on the conditional entropy-rates of such encodings when sources are correlated and Gaussian and the lattices involved are a from an asymptotically-well-behaved sequence. These upper bounds guarantee existence of a joint–compression stage which can increase encoder efficiency. The bounds exploit the property that the amount of possible values for one encoding collapses when conditioned on other sufficiently informative encodings. The bounds are applied to the scenario of communicating through a many-help-one network in the presence of strong correlated Gaussian interferers, and such a joint–compression stage is seen to compensate for some of the inefficiency in certain simple encoder designs.

## 1. Introduction

Lattice codes are a useful tool for information theoretic analysis of communications networks. Sequences of lattices can be designed to posess certain properties which make them useful for noisy channel coding or source coding in limit with dimension. These properties have been termed ‘good for channel coding’ and ‘good for source coding’ [1]. Sequences posessing both such properties exist, and an arbitrary number of sequences can be nested [2]. One application of ‘good’ sequences of nested lattices is in construction of distributed source codes for Gaussian signals. Well designed codes for such a scenario built off of such lattices enables encoders to produce a more efficient representation of their observations than would be possible without joint code design [3]. Such codes can provide optimal or near-optimal solutions to coding problems [4,5,6]. Despite their demonstrated ability to compress signals well in these cases, literature has identified redundancies across lattice encodings in other contexts [7,8,9,10]. In these cases, further compression of encodings is possible. This paper studies the correlation between lattice encodings of a certain design.

**Figure 1:**
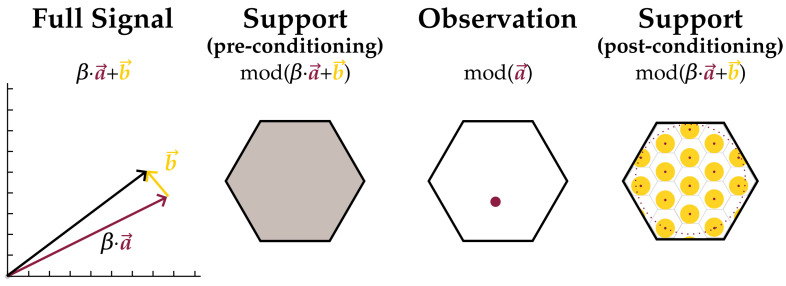
Collapse of the support of a random signal’s modulo after conditioning on the modulo of a related signal. Modulo is shown to some lattice *L* with base region *B*. Consider a signal comprised of two independent random components, $\vec{a}$ and $\vec{b}$, equaling $\beta \vec{a}+\vec{b}$. A possible outcome is drawn on the far left. Unconditioned, the support for $\mathrm{mod}(\beta \vec{a}+\vec{b})$ is the entire base region *B*, shown fully shaded in gray. Once $\mathrm{mod}(\vec{a})$ is observed, the component $\beta \vec{a}$ is known up to an additive factor in βL. If further the powers of $\vec{a}$ and $\vec{b}$ are bounded above, this leaves feasible points for $\mathrm{mod}(\beta \vec{a}+\vec{b})$ as a subset of those of the unconditioned variable. This subset is shaded yellow on the far right.

A class of upper bounds on the conditional Shannon entropies between lattice encodings of correlated Gaussian sources is produced by exploiting linear relations between lattice encodings and their underlying signals’ covariances. The key idea behind the analysis is that when the lattice-modulo of one random signal is conditioned on the lattice-modulo of a related signal, the region of feasible points for the first modulo collapses. A sketch of this support reduction is shown in Figure 1. This process is repeated until all information from the conditionals is integrated into the estimate of the support set. The upper bound establishes stronger performance limits for such coding structures since it demonstrates that encoders are able to convey the same encodings at lower messaging rates.

### 1.1. Contributions

The following novel contributions are provided:

- A class of upper bounds on conditional entropy-rates of appropriately designed lattice encoded Gaussian signals.
- An application of the bounds to the problem of point-to-point communication through a many-help-one network in the presence of interference. This strategy takes advantage of a specially designed transmitter codebook’s lattice structure.
- A numerical experiment demonstrating the behavior of these bounds. It is seen that a joint–compression stage can partially alleviate inefficiencies in lattice encoder design.

### 1.2. Background

The redundancy of lattice-modulo-encoded messages has been noticed before, usually in the context of the following many-help-one problem: many `helpers’ observe correlated Gaussian signals and forward messages to a decoder which is interested in recovering a linear combination of said signals. Towards this end, Wagner in [7] provides an upper and lower bound on conditional entropies such as those here for a case with two lattice encodings. Yang in [8] realized a similar compression scheme for such encodings using further lattice processing on them and presents an insightful `coset planes’ abstraction. It was further noticed by Yang in [9] that improvement towards the many-help-one problem is obtained by splitting helper messages into two parts: one part a coarse quantization of the signal, compressed across helpers via Slepian–Wolf joint–compression (these message parts corresponding to the ‘high bit planes’), and another a lattice-modulo-encoding representing signal details (corresponding to ‘low bit planes’). This paper extends these ideas to a general quantity of helpers, and treats a case where a single component of the observations is known to have lattice structure.

Most recently, a joint–compression scheme for lattice encodings called ‘Generalized Compute Compress and Forward’ was introduced in [10], towards coding for a multi-user additive white Gaussian noise channel where a decoder seeks to recover all user’s messages and is informed by helpers. The scheme in [10] makes use of concepts from [9]. In the scheme each lattice message is split into a combination of multiple components, each component from a different coset plane. Design of which coset planes are used yields different performance results. Section 3 in the present work follows along the same lines, although for a network with one user and where many interferers without codebook structure are also present.

Throughout the paper, terminology and basic lattice theory results are taken from [1]. The lattice encoders studied are built from an ensemble of nested lattices, all both ‘good for quantization’ (Rogers-good) and ‘good for coding’ (Poltyrev-good). Such a construction is provided in [2]. An algorithm from [3] is also used which takes as an argument the structure of some lattice modulo encodings and returns linear combinations of the underlying signals recoverable by a certain type of processing on such encodings. This algorithm is listed here as Stages*$\left(\cdot \right)$ and is shown in Appendix A.

### 1.3. Outline

The main theorem providing upper bounds on conditional entropies of lattice messages, along with an overview of its proof is stated in Section 2. The theorem is slightly strengthened for an application to the problem of communicating over a many-help-one network in Section 3. A numerical analysis of the bounds is given in Section 3.2. A conclusion and discussion on the bound’s remaining inefficiencies is given in Section 4. A table of notation is provided in Table 1. A key for the interpretation of significant named variables is given in Table 2.

## 2. Main Results

The main results are as follows:

**Theorem** **1.** *For covariance $\Sigma \in R^{K\times K}$, take ${\vec{X}}^{n}=\left(X_{1}^{n},\ldots ,X_{K}^{n}\right)$ to be n independent draws from the joint-distribution N(0,Σ). Take rates $r_{1},\ldots ,r_{K}>0$ and any ε>0. If n is large enough, an ensemble of nested lattices $L_{c}\subset \;L_{1},\ldots ,L_{K}$ (with base regions $B_{c}⊃B_{1},\ldots ,B_{K}$) from [2] (Theorem 1) can be designed so that the following holds. First fix independent dithers $W_{k}∼\mathrm{unif}B_{k}$. These dithers have $\mathrm{var}W_{k}=2^{-2r_{k}}.$ Also fix $Y_{k}:={\mathrm{round}}_{B_{k}}\left(X_{k}^{n}+W_{k}\right)-W_{k}$ and lattice modulo encodings $U_{k}:={\mathrm{mod}}_{B_{c}}\left({\mathrm{round}}_{B_{k}}\left(X_{k}^{n}+W_{k}\right)\right)$.*

*Now for any ${\vec{\alpha }}_{0}\in Z^{K-1}$, number $n_{0}\in N$, basis $\left\{{\vec{\alpha }}_{1},\ldots ,{\vec{\alpha }}_{K}\right\}\subset Z^{K},$ fix variables:*

$$\begin{array}{cc} Y_{0} & :=Y_{K}+\frac{1}{n_{0}}{\vec{\alpha }}_{0}^{†}{\vec{Y}}_{\left[K-1\right]},\\ {\vec{Y}}_{c} & :=(Y_{0}-Y_{K},Y_{1},\ldots ,Y_{K-1}),\\ \delta_{0}^{2} & :=n_{0}^{2},\\ \sigma_{k}^{2} & :=\mathrm{var}\left(Y_{0}|{STAGES}^{*}\;{\left(\mathrm{var}\left({\vec{Y}}_{c}|{\left({\vec{\alpha }}_{j}^{†}{\vec{Y}}_{c}\right)}_{0<j\leq k}\right)\right)}^{†}{\vec{Y}}_{c}\right),k\in \left\{0\right\}\cup \left[K\right],\\ \delta_{k}^{2} & :=\mathrm{var}\left({\vec{\alpha }}_{k}^{†}{\vec{Y}}_{c}|{STAGES}^{*}\;{\left(\mathrm{var}\left({\vec{Y}}_{c}|{\left({\vec{\alpha }}_{j}^{†}{\vec{Y}}_{c}\right)}_{0<j<k}\right)\right)}^{†}{\vec{Y}}_{c}\right),k\in \left[K\right]. \end{array}$$

*Then the conditional entropy-rate is bounded:*

$$\begin{array}{cc} \frac{1}{n}H\left({\vec{U}}_{K}|{\vec{U}}_{\left[K-1\right]},\vec{W}\right) & \leq \min_{k\in \{0\}\cup [K]}\left[r_{K}+\frac{1}{2}\log\sigma_{k}^{2}+\sum_{j=0}^{k}\max\left\{\frac{1}{2}\log\delta_{j}^{2},0\right\}\right]+K^{2}\cdot \varepsilon . \end{array}$$

*Bounds of this form hold simultaneously for any subset and reordering of message indices 1,…,K*.

Proof for Theorem 1 is given in Appendix B. The proof is built from [3] (Theorem 1), its associated algorithm ${STAGES}^{*}\;\left(\cdot \right)$ (listed here in Appendix A) and two lemmas which provide useful decompositions of the involved random variables.

**Lemma** **1.** *Take variables as in the statement of Theorem 1. Then, the ensemble of lattices described can include an ‘auxiliary lattice’ ${\hat{L}}^{\prime }\subset L_{K}$ with base region ${\hat{B}}^{\prime }$, nesting ratio $\frac{1}{n}\log\left|{\hat{B}}^{\prime }\cap L_{K}\right|\to \frac{1}{2}\log\sigma^{2}+\varepsilon$ so that*

$$\begin{array}{cc} U_{K} & ={\mathrm{mod}}_{B_{c}}\left(C_{}+\frac{1}{n_{0}}\tilde{Y}+{\tilde{Y}}_{⊥}\right), \end{array}$$

*where $C_{},D_{}$ are functions of $\left({\vec{U}}_{\left[K\right]},\vec{W}\right)$, and with high probability*

$$\begin{array}{cc} \tilde{Y} & =-{\vec{\alpha }}_{0}^{†}{\vec{Y}}_{\left[K-1\right]}\in \left(D_{}+L_{c}\right), \end{array}$$

$$\begin{array}{cc} {\tilde{Y}}_{⊥} & =E_{⊥}\;\left(Y_{0}|\vec{A}\right)\in \hat{B}, \end{array}$$

$$\begin{array}{cc} \vec{A} & ={STAGES}^{*}\;{\left(\mathrm{var}{\vec{Y}}_{c}\right)}^{†}{\vec{Y}}_{c}. \end{array}$$

*In addition, $\sigma^{2}=\max\left\{2^{-2r_{K}},\mathrm{var}{\tilde{Y}}_{⊥}\right\}.$*

**Lemma** **2.** Take variables as in the statement of Theorem 1. Then, the ensemble of lattices described can include ‘auxiliary lattices’ $\hat{L}\subset L_{c},{\hat{L}}^{\prime }\subset L_{K}$ with base regions $\hat{B},{\hat{B}}^{\prime }$, nesting ratios $\frac{1}{n}\log|\hat{B}\cap L_{c}|\to \frac{1}{2}\log\delta^{2}+\varepsilon ,\frac{1}{n}\log\left|{\hat{B}}^{\prime }\cap L_{K}\right|\to \frac{1}{2}\log\sigma^{2}+\varepsilon$ so that, for any linear combination Y of ${\vec{Y}}_{\left[K\right]}$, vector $\vec{\alpha }\in Z^{K}$, matrix $A\in R^{*\times K}$ and $\vec{A}=A{\vec{Y}}_{c}$, then

$$\begin{array}{c} Y=C_{}+\beta \tilde{Y}+{\tilde{Y}}_{⊥}, \end{array}$$

*where $C_{},D_{}$ are functions of $\left(\vec{A},{\mathrm{mod}}_{n_{0}B_{c}}\left(Y_{0}\right),{\vec{U}}_{\left[K\right]},\vec{W}\right)$, β is some scalar estimation coefficient, and with high probability*

$$\begin{array}{cc} \tilde{Y} & =E_{⊥}\;\left({\vec{\alpha }}^{†}Y_{c}|\vec{A}\right)\in \left(D_{}+L_{c}\right)\cap \hat{B}, \end{array}$$

$$\begin{array}{cc} {\tilde{Y}}_{⊥} & =E_{⊥}\;\left(Y|\vec{A},\tilde{Y}\right)\in {\hat{B}}^{\prime }. \end{array}$$

*In addition, $\delta^{2}=\mathrm{var}\tilde{Y}$, $\sigma^{2}=\max\left\{2^{-2r_{K}},\mathrm{var}{\tilde{Y}}_{⊥}\right\}$*.

Proofs for Lemmas 1, 2 are given in Appendix B. These lemmas do not strictly require that the sources be multivariate normal. This technical generalization is relevant in the application to the communication strategy in Section 3. Broadly, the proof of Theorem 1 goes as follows.

1. Choose some ${\vec{\alpha }}_{0}\in Z^{K-1},\;n_{0}\in N.$ Apply Lemma 1 to $U_{K}$. Call ${\tilde{Y}}_{⊥}$ a ‘residual.’
2. Choose some ${\vec{\alpha }}_{}\in Z^{K}.$ Apply Lemma 2 to the residual to break the residual ${\tilde{Y}}_{⊥}$ up into the sum of a lattice part due to ${\vec{\alpha }}_{}^{†}{\vec{Y}}_{\left[K-1\right]}$ and a new residual, whatever is left over.
3. Repeat the previous step until the residual vanishes (up to K-1 times). Notice that this process has given several different ways of writing $U_{K}$; by stopping at any amount of steps, $U_{K}$ is the modulo sum of several lattice components and a residual.
4. Design the lattice ensemble for the encoders such that the log-volume contributed to the support of $U_{K}$ by each component can be estimated. The discrete parts will each contribute log-volume $\frac{1}{2}\log\delta^{2}$ and residuals log-volume $r_{K}+\frac{1}{2}\log\sigma^{2}.$
5. Recognize the entropy of $U_{K}$ is no greater than the log-volume of its support. Choose the lowest support log-volume estimate of those just found.

Notice that each lemma application involves choice of some integer parameters. Choices which yield the strongest bound are unknown. Possible schemes for these decisions are the subroutines Alpha0(·), Alpha(·), listed in Appendix A. As implemented, Alpha0(·) chooses $n_{0}=1$ and the integer linear combination ${\vec{\alpha }}_{0}$ which leaves the least residual. As implemented, $ALPHA\;\left(\cdot \right)$ chooses the integer linear combination ${\vec{\alpha }}_{}$ for which ${\vec{\alpha }}_{}^{†}{\vec{Y}}_{\left[K-1\right]}$ is closest to being recoverable from current knowledge at each lemma application. It produces the combination for which the entropy $\frac{1}{2}\log\delta^{2}$ of the unknown part of ${\vec{\alpha }}_{}^{†}{\vec{Y}}_{\left[K-1\right]}$ is minimized. This may be a suboptimal choice since, while such combinations are close to recoverable, they may not be very pertinent to a description of $U_{K}$. Nonetheless, it is still a good enough rule to produce nontrivial entropy bounds, as seen in Section 3.2.

## 3. Lattice-Based Strategy for Communication via Decentralized Processing

**Figure 2:**
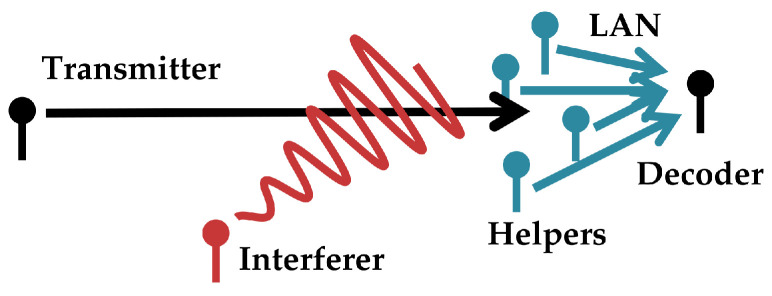
High level overview of the communications scenario in Section 3. A transmitter seeks to communicate digital information to a decoder through a Gaussian channel in the presence of Gaussian interference (one interferer drawn). The decoder is informed of the transmitter’s signal through helpers which pass it digital information through an out-of-band local area network (LAN).

**Figure 3:**
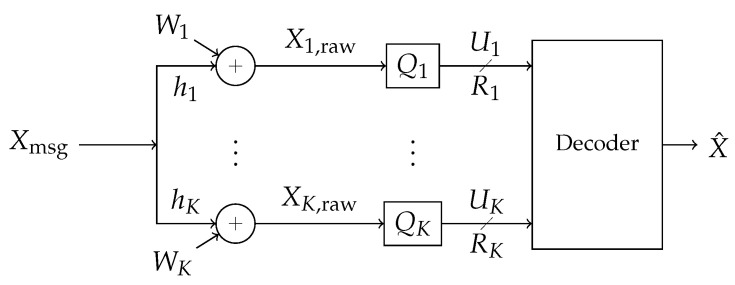
Block diagram of the communications scenario. A signal $X_{\mathrm{msg}}$ from a codebook is broadcast through an additive white Gaussian noise (AWGN) channel in the presence of independent Gaussian interference, creating correlated additive noise $\left(W_{1},\ldots ,W_{K}\right)$. The signal is observed at *K* receivers labeled $Q_{1},\ldots ,Q_{K}$. The *k*-th reciver observes $X_{k,\mathrm{raw}}$ and processes its observation into a rate-$R_{k}$ message $U_{k}$. The messages are forwarded losslessly over a local area side channel to a decoder which attempts to recover the message.

Consider a scenario where a decoder seeks to decode a message from a single-antenna broadcaster in an additive white Gaussian noise (AWGN). The decoder does not observe a signal directly but instead is provided information by a collection of distributed observers (‘helpers’) which forward it digital information, each observer-to-decoder link supporting a different communications rate. This network is depicted in Figure 2. A block diagram is shown in Figure 3. This is the problem of a single-antenna transmitter communicating to a decoder informed out-of-band by a network of helpers in the presence of additive white Gaussian noise and interference.

Note that this problem is different from the problem of distributed source coding of a linear function [3,7,8,9,11]. In contrast to the source coding problem, the signal being preserved by the many-help-one network in the present case has a codebook structure. This structure can be exploited to improve the source-to-decoder communications rate. This problem has been studied [12,13], but the best achievable rate is still unknown. In this section, we present a strategy that takes advantage of this codebook structure.

The core of the strategy is to apply a slight modification of Theorem 1 to the network. The transmitter modulates its communications message using a nested lattice codebook such as one in [4]. The helpers employ lattice encoders such as those from Theorem 1, and then perform Slepian–Wolf distributed lossless compression [14] (Theorem 10.3) on their encodings to further reduce their rate. Because the codeword appears as a component of all the helper’s observations, the bound on the message’s joint entropy obtained from Theorem 1 can be strengthened, allowing one to use a more aggressive compression stage.

### 3.1. Description of the Communication Scheme

It is well known that a nested lattice codebook with dither achieves Shannon information capacity in a point-to-point AWGN channel with a power-constrained transmitter [4]. One interesting aspect of the point-to-point communications scheme described in [4] is that decoding of the noisy signal is done in modulo space. We will see in this section how lattice encodings like those in Theorem 1 can be used to provide such a decoder enough information to recover a communications message.

Without loss of generality, assume that the transmitter is limited to have average transmission power 1. The scheme’s codebook is designed from nested lattices $L_{f,\mathrm{msg}}⊃L_{c,\mathrm{msg}}$ with base regions $B_{f,\mathrm{msg}},B_{c,\mathrm{msg}}$. $L_{f,\mathrm{msg}}$ is chosen to be good for coding and $L_{c,\mathrm{msg}}$ good for quantization. The messaging rate of this codebook is determined by the nesting ratio of $L_{c,\mathrm{msg}}$ in $L_{f,\mathrm{msg}}$:

$$R_{\mathrm{msg}}:=\frac{1}{n}\log\left|L_{f,\mathrm{msg}}\cap B_{c,\mathrm{msg}}\right|.$$

Lattices can be designed with nesting ratios such that any rate above zero can be formed. Taking a message $M\in L_{f,\mathrm{msg}}\cap B_{c,\mathrm{msg}}$ and choosing a dither $W_{\mathrm{msg}}∼-B_{c,\mathrm{msg}}$ of which the decoder is informed, then the codeword associated with *M* takes the form:

$$\begin{array}{c} X_{\mathrm{msg}}^{n}\left(M\right):=\frac{{\mathrm{mod}}_{L_{c,\mathrm{msg}}}\left(M+W_{\mathrm{msg}}\right)}{\sqrt{\mathrm{var}W_{\mathrm{msg}}}}\in \frac{B_{L_{c,\mathrm{msg}}}}{\sqrt{\mathrm{var}W_{\mathrm{msg}}}}\subset R^{n}. \end{array}$$

We now describe observations of such a signal by helpers in the presence of AWGN interferers. For covariance $\Sigma_{\mathrm{noise}}\in R^{K\times K}$, take

$${\vec{X}}_{\mathrm{noise}}^{n}=\left(X_{\mathrm{noise},1}^{n},\ldots ,X_{\mathrm{noise},K}^{n}\right)\in {\left(R^{n}\right)}^{K}$$

to be *n* independent draws from the joint-distribution $N(0,\Sigma_{\mathrm{noise}}).$ In addition, take a random vector $X_{\mathrm{msg}}^{n}$ as described at the beginning of Section 3.1 and a vector $c_{\mathrm{msg}}\in R^{K}$ and define $\Sigma_{\mathrm{msg}}:=c_{\mathrm{msg}}c_{\mathrm{msg}}^{†}$. Now, the *k*-th helper observes the vector:

$$X_{k}^{n}={\left[c_{\mathrm{msg}}\right]}_{k}X_{\mathrm{msg}}^{n}+X_{\mathrm{noise},k}^{n}\in R^{n}.$$

Form an observations vector:

$${\vec{X}}^{n}:=c_{\mathrm{msg}}\left(X_{\mathrm{msg}}^{n}\right)+{\vec{X}}_{\mathrm{noise}}^{n}\in {\left(R^{n}\right)}^{K},$$

and finally form a cumulative time-averaged covariance matrix as

$$\Sigma :=\mathrm{var}{\vec{X}}^{n}=c_{\mathrm{msg}}c_{\mathrm{msg}}^{†}+\Sigma_{\mathrm{noise}}\in R^{K\times K}.$$

If helpers are informed of message dither $W_{\mathrm{msg}}$, then they are informed of the codebook for $X_{\mathrm{msg}}$ and its lattice structure. Using lattice encoders such as those described in Theorem 1, this codebook information can be used to strengthen the upper bound on conditional entropies between the messages.

**Theorem** **2.** *In the context of the channel description given in Section 3.1, entropy bounds identical to those from Theorem 1 hold for its described observer encodings. The bounds also hold re-defining:*

$$\begin{array}{cc} Y_{0} & :=X_{msg}, \end{array}$$

*defining the rest of the variables in the theorem as stated. The bounds also hold instead re-defining:*

$$\begin{array}{cc} Y_{c} & :=(Y_{0}-Y_{K},Y_{1},\ldots ,Y_{K-1},X_{src}), \end{array}$$

*vectors $\left\{{\vec{\alpha }}_{1},\ldots ,{\vec{\alpha }}_{K+1}\right\}\subset Z^{K+1}$ a basis where all vectors but one ${\vec{\alpha }}_{s},s\in \left[K+1\right]$ have 0 as their (K+1)-th component and ${\vec{\alpha }}_{s}={\left[0,0,\ldots ,0,1\right]}^{†},$ taking*

$$\begin{array}{cc} {\vec{a}}_{R}^{\left(msg\right)} & \in \mathrm{image}{STAGES}^{*}\;\left(\mathrm{var}\left({\left[{\vec{Y}}_{c}\right]}_{\left[K\right]}|{\left({\vec{\alpha }}_{j}^{†}{\vec{Y}}_{c}\right)}_{0<j<s}\right)\right),\\ {\vec{a}}_{Z}^{\left(msg\right)} & \in Z^{K},\\ \lambda^{\left(msg\right)} & :=\mathrm{cov}(X_{\mathrm{msg}}^{n},{\left({\vec{a}}_{R}^{\left(msg\right)}+{\vec{a}}_{Z}^{\left(msg\right)}\right)}^{†}{\left[{\vec{Y}}_{c}\right]}_{\left[K\right]}),\\ Y_{⊥}^{\left(msg\right)} & :=E\;\left({\left({\vec{a}}_{R}^{\left(msg\right)}+{\vec{a}}_{Z}^{\left(msg\right)}\right)}^{†}{\left[{\vec{Y}}_{c}\right]}_{\left[K\right]}|X_{\mathrm{msg}}^{n}\right),\\ \delta_{\left(msg\right)}^{2} & :={\left(\frac{\lambda^{\left(msg\right)}}{\gamma_{n}}-1\right)}^{2}+\mathrm{var}Y_{⊥}^{\left(msg\right)},\\ \delta_{s}^{2} & :=\max\{1,\frac{\delta_{\left(msg\right)}^{2}}{2^{-2r_{msg}}}+\varepsilon \}, \end{array}$$

*and taking the rest of the variables in the theorem as stated over range k∈[K+1].*

A sketch for Theorem 2 is provided in Appendix C. The theorem’s statement can be broadly understood in terms of the proof of Theorem 1. After a number of steps *s* in the support analysis for Theorem 1, the codebook component $X_{msg}^{n}$ can be partially decoded yielding tighter estimation of that component’s contribution to the support of $U_{K}$. The variables $\lambda^{\left(msg\right)},{\vec{a}}_{R}^{\left(msg\right)},{\vec{a}}_{Z}^{\left(msg\right)}$ are parameters for this partial decoding. Lattice modulo messages such as those described in Theorem 2 can be recombined in a useful way:

**Lemma** **3.** For ε>0 and vectors $a_{Z}\in Z^{K}$, $a_{R}\in \mathrm{image}{STAGES}^{*}\;\left(\Sigma \right)\subset R^{K}$, then lattice modulo encodings ${\vec{U}}_{\left[K\right]}$ from Theorem 2 can be processed into:

(1) $$U_{\mathrm{proc}}:={\mathrm{mod}}_{L_{c,\mathrm{msg}}}\left(\lambda X_{\mathrm{msg}}+Y_{\mathrm{noise}}\right),$$

*where λ∈R is some constant:*

$$\lambda :=\mathrm{cov}\left(X_{\mathrm{msg}}^{n},{\left(a_{Z}+a_{R}\right)}^{†}{\vec{Y}}_{\left[K\right]}\right)$$

*and the noise term $Y_{\mathrm{noise}}$ has the following properties:*

- *$\sigma_{\mathrm{noise}}^{2}:=\mathrm{var}Y_{\mathrm{noise}}=\mathrm{var}\left({\left(a_{Z}+a_{R}\right)}^{†}{\vec{Y}}_{\left[K\right]}|X_{\mathrm{msg}}^{n}\right),$*
- $Y_{\mathrm{noise}}⊥\left(X_{\mathrm{msg}},M,W_{\mathrm{msg}}\right),$
- *$Y_{\mathrm{noise}}$ is with high probability in the base cell of any lattice good for coding semi norm-ergodic noise up to power*$\sigma_{\mathrm{noise}}^{2}+\varepsilon$.

Lemma 3 is demonstrated in Appendix D. Notice that Equation (1) is precisely the form of signal processed by the communications decoder described in [4]. The following result summarizes the performance of this communications strategy.

**Corollary** **1.** *Fix a codebook rate $r_{\mathrm{msg}}>0.$ As long as helper-to-decoder messaging rates $R_{1},\ldots ,R_{K}>0$ satisfy all the following criteria:*

(2) $$\forall S\subset \left[K\right],\;\sum_{k\in S}R_{k}>\tilde{H}\left(S|\left[K\right]\backslash S\right)+\varepsilon ,$$

*each $\tilde{H}\left(S|\left[K\right]\backslash S\right)$ being any entropy-rate bound obtained from Theorem 2, then the following communications rate from source to decoder is achievable, taking $a_{Z},a_{R},\lambda ,\sigma_{\mathrm{noise}}^{2}$ from their definitions in Lemma 3:*

(3) $$R_{\mathrm{msg}}<\min\left\{r_{\mathrm{msg}},\;\underset{a_{Z},\;a_{R}}{\sup}\max_{\gamma^{2}\in \left(0,1\right]}\frac{1}{2}\log\left[\frac{\gamma^{2}}{{\left(\lambda -\gamma \right)}^{2}+\sigma_{\mathrm{noise}}^{2}}\right]\right\}.$$

Proof for Corollary 1 is given in Appendix E, and evaluation of the achieved communications rates for certain lattice code designs is shown in Section 3.2.

### 3.2. Numerical Results

The achievable rate given in Corollary 1 depends on the design of the lattice encoding scheme at the helpers. Identification of the best such lattice encoders for such a system is closely tied to a receivers’ covariance structure [3]. For this reason and for the purpose of evaluating the effect of joint compression stage, we restrict our attention to a particular channel structure and lattice encoder design.

**Figure 4:**
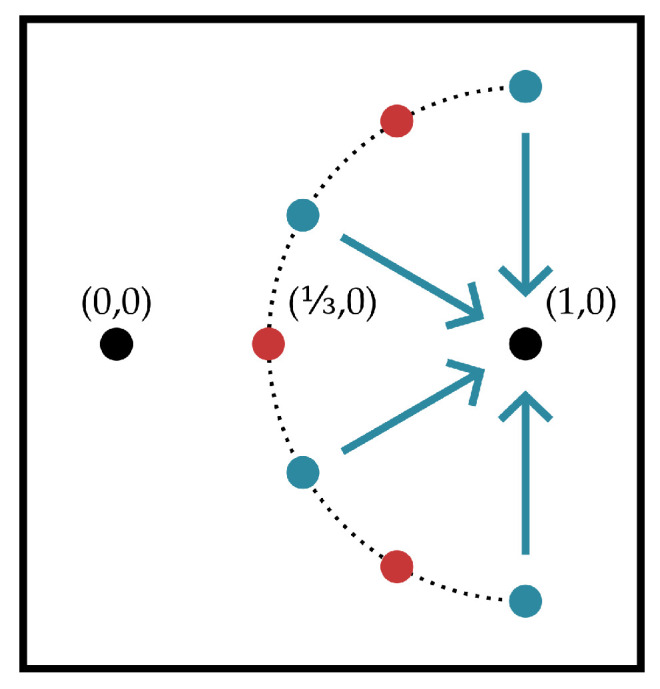
The line-of-sight channel considered. A black transmit node at (0,0) seeks to communicate with a black decoder node at (1,0). Three red ‘interferer’ nodes broadcast an independent Gaussian signal, each interferer has its own signal. The decoder does not observe any signal directly but is forwarded messages from four blue ‘helper’ nodes which observe signals through a line-of-sight additive-white-Gaussian noise channel. The interferers and helpers are oriented alternatingly and equispaced about a radius-2/3 semicircle towards the encoder with center (1,0).

The line-of-sight configuration shown in Figure 4 is considered. It yields helper observations with the following covariance structure, labeling interferer signals in Figure 4 from top to bottom as $\left(W_{I1},W_{I2},W_{I3}\right)$ and indexing helpers from top to bottom:

(4) $$\begin{array}{cc} X_{raw,1} & =\frac{\sqrt{P_{S}}}{∥1+\left(\frac{2}{3}\right)e^{i\pi \cdot 1/2}∥}X_{msg}+W_{1}+\ldots \\ & +\frac{\sqrt{P_{I}}}{∥\left(\frac{2}{3}\right)\left(e^{i\pi \cdot 1/2}-e^{i\pi \cdot 2/3}\right)∥}W_{I1}+\frac{\sqrt{P_{I}}}{∥\left(\frac{2}{3}\right)\left(e^{i\pi \cdot 1/2}-e^{i\pi \cdot 1}\right)∥}W_{I2}+\frac{\sqrt{P_{I}}}{∥\left(\frac{2}{3}\right)\left(e^{i\pi \cdot 1/2}-e^{i\pi \cdot 4/3}\right)∥}W_{I3},\\ X_{raw,2} & =\frac{\sqrt{P_{S}}}{∥1+\left(\frac{2}{3}\right)e^{i\pi \cdot 5/6}∥}X_{msg}+W_{2}+\ldots \\ & +\frac{\sqrt{P_{I}}}{∥\left(\frac{2}{3}\right)\left(e^{i\pi \cdot 5/6}-e^{i\pi \cdot 2/3}\right)∥}W_{I1}+\frac{\sqrt{P_{I}}}{∥\left(\frac{2}{3}\right)\left(e^{i\pi \cdot 5/6}-e^{i\pi \cdot 1}\right)∥}W_{I2}+\frac{\sqrt{P_{I}}}{∥\left(\frac{2}{3}\right)\left(e^{i\pi \cdot 5/6}-e^{i\pi \cdot 4/3}\right)∥}W_{I3},\\ X_{raw,3} & =\frac{\sqrt{P_{S}}}{∥1+\left(\frac{2}{3}\right)e^{i\pi \cdot 7/6}∥}X_{msg}+W_{3}+\ldots \\ & +\frac{\sqrt{P_{I}}}{∥\left(\frac{2}{3}\right)\left(e^{i\pi \cdot 7/6}-e^{i\pi \cdot 2/3}\right)∥}W_{I1}+\frac{\sqrt{P_{I}}}{∥\left(\frac{2}{3}\right)\left(e^{i\pi \cdot 7/6}-e^{i\pi \cdot 1}\right)∥}W_{I2}+\frac{\sqrt{P_{I}}}{∥\left(\frac{2}{3}\right)\left(e^{i\pi \cdot 7/6}-e^{i\pi \cdot 4/3}\right)∥}W_{I3},\\ X_{raw,4} & =\frac{\sqrt{P_{S}}}{∥1+\left(\frac{2}{3}\right)e^{i\pi \cdot 3/2}∥}X_{msg}+W_{4}+\ldots \\ & +\frac{\sqrt{P_{I}}}{∥\left(\frac{2}{3}\right)\left(e^{i\pi \cdot 3/2}-e^{i\pi \cdot 2/3}\right)∥}W_{I1}+\frac{\sqrt{P_{I}}}{∥\left(\frac{2}{3}\right)\left(e^{i\pi \cdot 3/2}-e^{i\pi \cdot 1}\right)∥}W_{I2}+\frac{\sqrt{P_{I}}}{∥\left(\frac{2}{3}\right)\left(e^{i\pi \cdot 3/2}-e^{i\pi \cdot 4/3}\right)∥}W_{I3},\\ W_{k} & ∼N(0,1)\;i.i.d. \end{array}$$

where $P_{S},P_{I}>0$ are signal, interferer powers, respectively. Choice of this channel is arbitrary but provides an instance where the decoder would not be able to recover the signal of interest if it observed directly without the provided helper messages.

#### 3.2.1. Communications Schemes

First, we describe a class of lattice encoders the four helpers could employ:

- Fix some c∈(0,3). If helper k∈[4] in the channel from Figure 4 observes $X_{\mathrm{raw},k}^{n}$, then it encodes a normalized version of the signal:
  $$X_{k}^{n}:=\frac{c}{\sqrt{\mathrm{var}X_{\mathrm{raw},k}^{n}}}X_{\mathrm{raw},k}^{n}.$$
- Fix equal lattice encoding rates per helper $r=r_{1}=r_{2}=r_{3}=r_{4}$, and take lattice encoders as described in Theorem 1. Note that these rates may be distinct from the helper-to-base rates $R_{1},\ldots ,R_{4}$ if post-processing of the encodings is involved.

**Figure 5:**
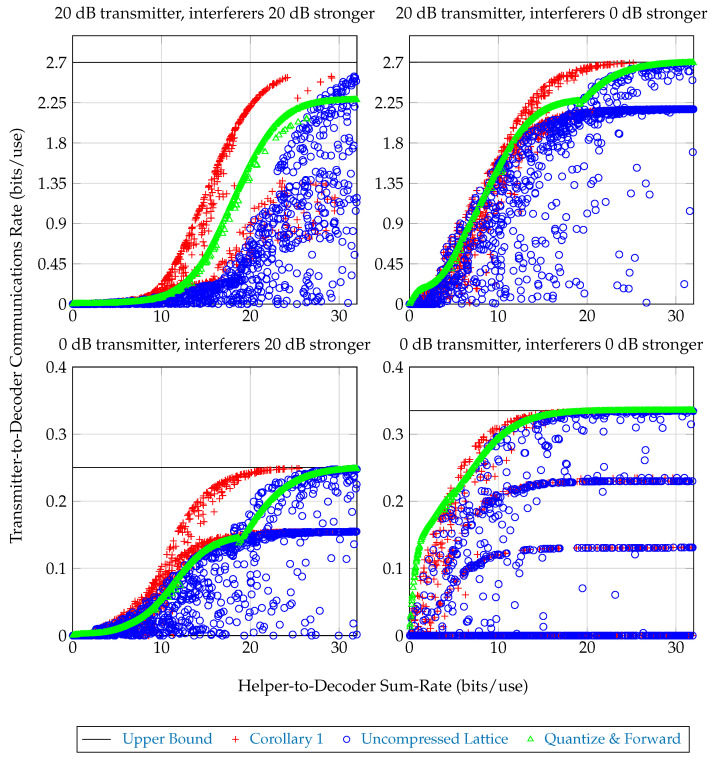
Communications rate versus helper-sum-rate for 1000 randomly chosen encoding schemes as described in Section 3.2.1 in the line-of-sight channel from Figure 4, Equation (4). In each subplot, the transmitter broadcasts with power such that the average SNR seen across helpers is the given ‘transmitter’ dB figure. Each interferer broadcasts its own signal with its power the given ‘interferer’ dB stronger than the transmitter’s power. Notice that, although the uncompressed lattice scheme is often outperformed by plain Quantize & Forward for the same helper message rates, adding a properly configured compression stage can more than make up for the sum-rate difference. In certain regimes, even the compressed lattice scheme performs worse or practically the same as Quantize & Forward, indicating the given lattice encoder design is weak; uncompressed lattice encoders can be configured to implement the Quantize & Forward scheme.

Communications schemes involving lattice encoders of this form are compared in Figure 5 over an ensemble of choices for lattice encoder rates *r* and scales c∈(0,3). Achieved transmitter-to-decoder communication rate versus sum-rate from helpers to decoder are plotted. The following quantities are plotted:

- *Upper Bound*: An upper bound on the achievable transmitter-to-decoder communications rate, corresponding to helpers which forward with infinite rate. This bound is given by the formula $I(X_{msg};{\left(X_{\mathrm{raw},k}\right)}_{k\in [4]})$.
- *Corollary 1* The achievable communications rate from Corollary 1, where each helper computes the lattice encoding described above, then employs a joint–compression stage to reduce its messaging rate. The sum-helpers-to-decoder rate for this scheme is given by Equation (2), taking S=[4]. The achieved messaging rate is given by the right-hand-side of Equation (3).
- *Uncompressed Lattice*: The achievable communications rate from Corollary 1, with each helper forwarding to the decoder its entire lattice encoding without joint–compression. The sum-helpers-to-decoder rate for this scheme is 4r since in this scheme each helper forwards to the base at rate $R_{k}=r$. The achieved messaging rate is given by the right-hand-side of Equation (3).
- *Quantize & Forward*: An achievable communications rate where helper-to-decoder rates $R_{k},k\in \left[4\right]$ are chosen so that $R_{1}+R_{2}+R_{3}+R_{4}=R_{sum}$ and each helper forwards a rate-distortion-optimal quantization of its observation to the decoder. The decoder processes these quantizations into an estimate of $X_{\mathrm{msg}}$ and decodes. This is discussed in more detail in [13]. The sum-helpers-to-decoder rate for this scheme is $R_{sum}$. The achieved messaging rate is $I(X_{msg};{\left(X_{raw,k}+Z_{k}\right)}_{k\in [4]})$, where $Z_{k}∼N\left(0,\mathrm{var}\left(X_{raw,k}\right)\cdot 2^{-2R_{k}}\right)$.

Performance of these strategies for different broadcaster powers is shown in Figure 5. It is seen that, although the lattice encoder designs are poor, the joint–compression stage partially compensates for this, and with joint compression the scheme outperforms the plain ‘Quantize & Forward’ scheme. Notice that none of the strategies produce convex rate regions, indicating that time-sharing can be used to achieve better rates in some regimes.

In all figures shown, the gap between achieved rates from the joint–compression bound given from Theorem 1 and Theorem 2 (the latter being an improvement) were often nonzero but too small to noticeably change the graphs in Figure 5. For this reason only, achievable rates for the strategy from Corollary 1 are plotted. The gain from involving codebook knowledge in lattice encoding compression is either insignificant for the tested scenario, or choices in computing the upper bounds are too poor to reveal its performance gains. Sub-optimality of the algorithm implementations here are all summarized and discussed in Section 4.

## 4. Conclusions

A class of upper bounds on the joint entropy of lattice-modulo encodings of correlated Gaussian signals was presented in Theorem 1. Proof of these bounds involves reducing the problem to the entropy of one lattice message, say, $U_{K}$ conditioned on the rest, ${\vec{U}}_{\left[K-1\right]}$. The upper bound for this reduced case involves an iterative construction where in each step a suitable integer vector is chosen. Choice of vectors in these steps determines the order in which the observed lattice-modulo components are integrated into an estimate of $U_{K}$’s support. Different choice of vectors at each step yields a different bound, and the strongest sequence of choices is unknown. For numerical results in Section 3.2, a certain suboptimal was used although there is no guarantee that this choice is optimal.

The upper bounds were applied to the problem of communicating through a many-help-one network, and these bounds were evaluated for a rendition of the problem using lattice codes of simple structure. The bounds in Theorem 1 can be strengthened in this scenario by integrating codebook knowledge. This strengthening is described in Theorem 2.

In spite of the suboptimal lattice encoder designs analyzed, it was seen in Section 3.2 that jointly-compressed lattice encoders are able to significantly outperform more basic schemes in the presence of heavy interference, even when the joint compression stage uses the weaker entropy bounds from Theorem 1. In the numerical experiments tried, the strengthening in Theorem 2 was not seen to significantly improve compression. Whether this is typically true or just an artifact of poor design of the joint-compression stage is unknown. In either case, the simpler joint-compression strategy without codebook knowledge was seen to improve performance.

The most immediate forwards steps to the presented results is in characterization of the search problems posed by Theorems 1, 2. Although not discussed, corner-points of joint compression described here are implementable using further lattice processing on the encodings $U_{1},\ldots ,U_{K}$ and their dithers $\vec{W}$. Such a process might mimic the compression procedure described in [10]. Tightness arguments from this work may also apply to the present less structured channel.

Finally, according to the transmission method in [10], the achievable rate in Corollary 1 may be improvable by breaking the transmitter’s message *M* up into a sum of multiple components, each from a finer lattice. Joint–compression for such a transmission could integrate codebook information from each message component separately, allowing for more degrees of freedom in the compression stage’s design, possibly improving the achievable rate. This is an extension of the argument in Appendix D. These improvements are out of this paper’s scope but provide meaningful paths forward.

## Figures and Tables

**Table 1:** Symbols and notation.

a:=b	Define *a* to equal *b*
[n]	Integers from 1 to *n*
$A,\vec{a},\vec{A}$	Matrix, column vector, vector, random vector
$A^{†},{\vec{a}}^{†}$	Transpose (All matrices involved are real)
${\left[A\right]}_{S,T}$	Submatrix corresponding to rows *S*, columns *T* of A
${\vec{Y}}_{S}$	an |S|-vector, the sub-vector of $\vec{Y}$ including components with indices in *S*. If *S* has order then this vector respects *S*’s order.
$I_{K}$	K×K identity matrix
$0_{K}$	K×1 zero vector
$\mathrm{diag}\vec{a}$	Square diagonal matrix with diagonals $\vec{a}$
pinv(·)	Moore-Penrose pseudoinverse
N(0,Σ)	Normal distribution with zero mean, covariance Σ
X∼f	*X* is a random variable distributed like *f*
$X^{n},f\left(x^{n}\right)$	Vector of *n* independent trials of a random variable distributed like *X*, a function whose input is intended to be such a variable
var(a)	Variance (or covariance matrix) of (components of) *a*, averaged over time index.
var(a|b)	Conditional variance (or covariance matrix) of (components of) *a* given observation *b*, averaged over time index.
$\mathrm{cov}\left(a,b\right),\mathrm{cov}\left(a,b|c\right)$	Covariance between *a* and b,, covariance between *a* and *b* conditioned on *c*, averaged over time index.
$E\;\left(a|b\right)$	Linear MMSE estimate of *a* given observations *b*
$E_{⊥}\;\left(a|b\right)$	Complement of $E\;\left(a|b\right)$, i.e., $E_{⊥}\;\left(a|b\right):=a-E\;\left(a|b\right)$. An important property is that $E\;\left(a|b\right)$ and $E_{⊥}\;\left(a|b\right)$ are uncorrelated.
${\mathrm{round}}_{L}\left(\cdot \right),{\mathrm{mod}}_{L}\left(\cdot \right)$	Lattice round, modulo to a lattice *L* (when it is clear what base region is associated with *L*).

**Table 2:** Description of variables.

*K*	Number of lattice encodings in current context.
*n*	Scheme blocklength
$X_{k}^{n}$	Observation at receiver *k*
$W_{k}$	Lattice dither *k*
$U_{k}$	Lattice encoding *k*
$Y_{k}$	Quantization of $X_{k}^{n}$
${\vec{Y}}_{c}$	Ensemble of lattice quantizations, sans modulo
Σ	K×K time-averaged covariance between observations $X_{1}^{n},\ldots ,X_{K}^{n}$
$\Sigma_{Q}$	K×K time-averaged covariance between quantizations $Y_{1},\ldots ,Y_{K}$
$r_{1},\ldots ,r_{K}$	Nesting ratios for coarse lattice $L_{c}$ in the fine lattices $L_{1},\ldots ,L_{K}$, equivalent to the encoding rates of lattice codes when joint compression is not used
$R_{1},\ldots ,R_{K}$	Messaging rates for helpers in the Section 3 communications scenario
$r_{\mathrm{msg}}$	Nesting ratio for codebook coarse lattice $L_{c,\mathrm{msg}}$ in codebook fine lattice $L_{f,\mathrm{msg}}$ in Section 3, equivalent to codebook rate
${\vec{h}}_{\mathrm{msg}}$	Covariance between codeword and quantizations in Section 3
${\vec{\alpha }}_{s}$	Integer combination of ${\vec{Y}}_{c}$ to analyze in step *s* of Appendix B
$\delta_{s}^{2}$	Variance of ${\vec{\alpha }}_{s}^{†}{\vec{Y}}_{c}$ after removing prior knowledge in Appendix B
$\sigma_{s}^{2}$	Variance of $Y_{K}$ uncorrelated with prior knowledge and ${\vec{\alpha }}_{s}^{†}{\vec{Y}}_{c}$ in Appendix B
$\beta_{s}$	Regression coefficient for ${\vec{\alpha }}_{s}^{†}{\vec{Y}}_{c}$ in $Y_{K}$ after including prior knowledge at step *s* in Appendix B

## Appendix A. Subroutines

Here, we provide a list of subroutines involved in a statement of the results:

- Stages*(·) is a slight modification of an algorithm from [3], reproduced here in Algorithm 1. The original algorithm characterizes the integral combinations $A^{†}\vec{Y}$ which are recoverable with high probability from lattice messages $\vec{U}$ and dithers $\vec{W}$, excluding those with zero power. The exclusion is due to the algorithm’s use of $\mathrm{SLVC}\;\left(\cdot \right)$ as just defined. Such linear combinations never arose in the context of [3], although it provides justification for them being recoverable; in the paper, the algorithm’s argument is always full-rank. This is not true in the present context. The version here includes these zero-power subspaces by including a call to $LATTICEKERNEL\;\left(\cdot \right)$ before returning.
- $\mathrm{SLVC}\;\left(B\right)$, ‘Shortest Lattice Vector Coordinates’ returns the nonzero integer vector $\vec{a}$ which minimizes the norm of $B\vec{a}$ while $B\vec{a}\neq 0$, or the zero vector if no such vector exists. $\mathrm{SLVC}\;\left(\cdot \right)$ can be implemented using a lattice enumeration algorithm like one in [15] together with the LLL algorithm to convert a set of spanning lattice vectors into a basis [16].
- LatticeKernel(*B*,*A*), for $B\in R^{K\times d},\;A\in Z^{d\times a}$ returns the integer matrix $A_{⊥}\in Z^{d\times b}$ whose columns span the collection of all $\vec{a}\in Z^{K}$ where $B\vec{a}=0$ while $A^{†}\vec{a}=0_{a}$. In other words, it returns a basis for the integer lattice in kerB whose components are orthogonal to the lattice A. This can be implemented using an algorithm for finding ‘simultaneous integer relations’ as described in [17].
- $\mathrm{ICQM}\;\left(M,\vec{v},c\right)$ is an “Integer Convex Quadratic Minimizer.” It provides a solution for the NP-hard problem: “Minimize $\left({\vec{x}}^{†}M\vec{x}+2{\vec{v}}^{†}\vec{x}+c\right)$ over $\vec{x}$ with integer components.” Although finding the optimal solution is exponentially difficult in input size, algorithms are tractable for low dimension. [18] (Algorithm 5, Figure 2).
- CVarComponents$\left(\Sigma_{Q},A\right)$ returns certain variables $\left\{M,\vec{v},c\right\}$ involved in computing
  $$\mathrm{var}\left(Y_{K}-{\vec{\alpha }}_{}^{†}{\vec{Y}}_{\left[K-1\right]}|A{\vec{Y}}_{\left[K-1\right]}\right)$$
  when $\vec{Y}=\left(Y_{1},\ldots ,Y_{K}\right)$ has covariance $\Sigma_{Q}$. Write some matrices in block form:
  $$\begin{array}{cc} \Sigma_{Q} & =\left[\begin{array}{cc} M_{1} & {\vec{v}}_{1}\\ {\vec{v}}_{1}^{†} & \varsigma_{1}^{2} \end{array}\right],\\ \Sigma_{Q}\left[\begin{array}{c} A\\ 0 \end{array}\right]{\left({\left[\begin{array}{c} A\\ 0 \end{array}\right]}^{†}\Sigma_{Q}\left[\begin{array}{c} A\\ 0 \end{array}\right]\right)}^{-1}{\left[\begin{array}{c} A\\ 0 \end{array}\right]}^{†}\Sigma_{Q} & =\left[\begin{array}{cc} M_{2} & {\vec{v}}_{2}\\ {\vec{v}}_{2}^{†} & \varsigma_{2}^{2} \end{array}\right]. \end{array}$$
  Then, taking $M=\left(M_{1}-M_{2}\right),\;v=-\left({\vec{v}}_{1}-{\vec{v}}_{2}\right),\;c=\left(\varsigma_{1}^{2}-\varsigma_{2}^{2}\right),$ one can check that:
  $$\mathrm{var}\left(Y_{K}-{\vec{\alpha }}_{}^{†}{\vec{Y}}_{\left[K-1\right]}|A{\vec{Y}}_{\left[K-1\right]}\right)={\vec{\alpha }}_{}^{†}M{\vec{\alpha }}_{}+2{\vec{v}}^{†}{\vec{\alpha }}_{}+c.$$
- CVar$\left(M_{1}|M_{2};\Sigma \right)$ computes the conditional covariance matrix of $M_{1}^{†}\vec{Z}$ conditioned on $M_{2}^{†}\vec{Z}$ for $\vec{Z}∼N\left(0,\Sigma \right)$. This is given by the formula:
  $$CVAR\;\left(M_{1}|M_{2};\Sigma \right):=M_{1}^{†}\Sigma M_{1}-M_{1}^{†}\Sigma M_{2}\mathrm{pinv}\left(M_{2}^{†}\Sigma M_{2}\right)M_{2}^{†}\Sigma M_{2}.$$
- $ALPHA0\;\left(\Sigma_{Q},A\right)$ in Algorithm 2 implements a strategy for choosing ${\vec{\alpha }}_{0}$ in Theorems 1, 2.
- $ALPHA\;\left(\Sigma ,A\right)$ in Algorithm 3 implements a strategy for choosing ${\vec{\alpha }}_{s}$ in theorems 1, 2.

**Algorithm 1** Compute recoverable linear combinations $A\in R^{K\times m}$ from modulos of lattice encodings with covariance $\Sigma_{Q}\in R^{K\times K}$.
**function** Stages${}^{*}$(Σ) $A\leftarrow \left[\;\right],\;\vec{a}\leftarrow \mathrm{SLVC}\;\left(\Sigma_{Q}^{1/2}\right),\;R\leftarrow I_{K},$ **while** $0<{\left(R\vec{a}\right)}^{†}\Sigma_{Q}\left(R\vec{a}\right)<1$ **do** $A\leftarrow [A,\vec{a}]$ $R\leftarrow I_{K}-A\mathrm{pinv}\left(A^{†}\Sigma_{Q}A\right)A^{†}\Sigma_{Q}$ $\vec{a}\leftarrow \mathrm{SLVC}\;\left(\Sigma_{Q}^{1/2}R\right)$ **end while** $A\leftarrow [A,LATTICEKERNEL\;\left(\Sigma_{Q}^{1/2}R,A\right)]$ **return** A **end** **function**

**Algorithm 2** Strategy for choosing ${\vec{\alpha }}_{0}$ for Theorems 1, 2
**function** Alpha0(Σ) ▹ Find ${\vec{\alpha }}_{0}$ which minimizes $\mathrm{var}\left(Y_{K}-{\vec{\alpha }}_{0}^{†}{\vec{Y}}_{\left[K-1\right]}|A{\vec{Y}}_{\left[K-1\right]}\right)$ for $\Sigma =\mathrm{var}{\vec{Y}}_{\left[K-1\right]},A={STAGES}^{*}\;\left(\Sigma \right).$ $A\leftarrow {STAGES}^{*}\;\left(\Sigma \right).$ $\left\{M,\vec{v},c\right\}\leftarrow CVARCOMPONENTS\;\left(\Sigma ,A\right)$ $\left\{{\vec{\alpha }}_{},\sigma^{2}\right\}\leftarrow \mathrm{ICQM}\;\left(M,\vec{v},c\right)$ $n_{0}\leftarrow 1$ **return** $\left\{n_{0},{\vec{\alpha }}_{},\sigma^{2}\right\}$ **end** **function**

**Algorithm 3** Strategy for picking ${\vec{\alpha }}_{s}$ for Theorems 1, 2.
**function** Alpha(Σ,A) ▹*Entropy-greedy implementation: choose ${\vec{\alpha }}_{}$ where the unknown part of ${\vec{\alpha }}_{}^{†}{\vec{Y}}_{\left[K-1\right]}$ has the least entropy among any combination with an unknown part. Expects $\Sigma =\mathrm{var}{\vec{Y}}_{c},\;A={STAGES}^{*}\;\left(\mathrm{var}\left({\vec{Y}}_{c}|{\left[{\vec{\alpha }}_{0},\ldots ,{\vec{\alpha }}_{s-1}\right]}^{†}{\vec{Y}}_{c}\right)\right)$.* **if** rankA=K **then** ${\vec{\alpha }}_{}\leftarrow 0$ **else** $\Sigma_{\mathrm{reduced}}\leftarrow CVAR\;\left(\left.I_{K}|A;\;\Sigma \right.\right),\;{\vec{\alpha }}_{}\leftarrow \mathrm{SLVC}\;\left(\Sigma_{\mathrm{reduced}}\right)$ **end if** **return** ${\vec{\alpha }}_{}$ **end** **function**

## Appendix B. Proof of Lemmas 1, 2, Theorem 1

**Proof.** (Lemma 1) Take $D_{}:={\mathrm{mod}}_{B_{c}}\left({\vec{\alpha }}_{0}^{†}\left({\vec{U}}_{\left[K-1\right]}-{\vec{W}}_{\left[K-1\right]}\right)\right)$. Then, by modulo’s distributive property $D_{}={\mathrm{mod}}_{B_{c}}\left({\vec{\alpha }}_{0}^{†}{\vec{Y}}_{\left[K-1\right]}\right)$ so that $\tilde{Y}=-{\vec{\alpha }}_{0}^{†}{\vec{Y}}_{\left[K-1\right]}\in \left(D_{}+L_{c}\right)$. Compute:

$$\begin{array}{cc} \frac{1}{n_{0}}{\mathrm{mod}}_{n_{0}B_{c}}\tilde{Y} & =\frac{1}{n_{0}}{\mathrm{mod}}_{n_{0}B_{c}}\left(-{\vec{\alpha }}_{0}^{†}{\vec{Y}}_{\left[K-1\right]}\right). \end{array}$$

$$\begin{array}{cc} \; & ={\mathrm{mod}}_{B_{c}}\left(-\frac{1}{n_{0}}{\vec{\alpha }}_{0}^{†}{\vec{Y}}_{\left[K-1\right]}\right). \end{array}$$

Now:

$$\begin{array}{cc} U_{K} & ={\mathrm{mod}}_{B_{c}}\left(W_{k}+Y_{k}+\frac{1}{n_{0}}{\vec{\alpha }}_{0}^{†}{\vec{Y}}_{\left[K-1\right]}-\frac{1}{n_{0}}{\vec{\alpha }}_{0}^{†}{\vec{Y}}_{\left[K-1\right]}\right)\\ \; & ={\mathrm{mod}}_{B_{c}}\left(W_{k}+Y_{0}-\frac{1}{n_{0}}{\vec{\alpha }}_{0}^{†}{\vec{Y}}_{\left[K-1\right]}\right)\\ \; & ={\mathrm{mod}}_{B_{c}}\left(W_{k}+E\;\left(Y_{0}|\vec{A}\right)+E_{⊥}\;\left(Y_{0}|\vec{A}\right)-\frac{1}{n_{0}}{\vec{\alpha }}_{0}^{†}{\vec{Y}}_{\left[K-1\right]}\right)\\ \; & ={\mathrm{mod}}_{B_{c}}\left(W_{k}+E\;\left(Y_{0}|\vec{A}\right)+{\tilde{Y}}_{⊥}+\frac{1}{n_{0}}\tilde{Y}\right). \end{array}$$

By [3] (Theorem 1), $\vec{A}$ can be recovered by processing $\left({\vec{U}}_{\left[K-1\right]},\vec{W},\tilde{Y}\right)$, hence $E\;\left(Y_{0}|\vec{A}\right)$ can also be recovered. Choose $C_{}:=-W_{k}+E\;\left(Y_{0}|\vec{A}\right)$ so that the claim holds applying modulo’s distributive property. □

**Proof.** (Lemma 2) Take $U_{0}=n_{0}U_{K}+{\vec{\alpha }}_{0}^{†}U_{\left[K-1\right]},\;W_{0}=n_{0}W_{K}+{\vec{\alpha }}_{0}^{†}W_{\left[K-1\right]},\;{\vec{U}}_{c}=\left(U_{0},{\vec{U}}_{\left[K\right]}\right),{\vec{W}}_{c}=\left(W_{0},\vec{W}\right)$. Take $C_{}:=E\;\left({\vec{\alpha }}_{}^{†}{\vec{Y}}_{c}|{\vec{A}}_{}\right)$ and $D_{}:={\mathrm{mod}}_{B_{c}}\left({\vec{\alpha }}_{}^{†}\left({\vec{U}}_{c}-{\vec{W}}_{c}\right)-E\;\left({\vec{\alpha }}_{}^{†}Y_{c}|{\vec{A}}_{}\right)\right)={\mathrm{mod}}_{B_{c}}\left(E_{⊥}\;\left({\vec{\alpha }}_{}^{†}Y_{c}|{\vec{A}}_{}\right)\right)$. Choose $\beta :=\frac{\mathrm{cov}\left(Y,\tilde{Y}|{\vec{A}}_{}\right)}{\delta^{2}}$. Include good-for-coding auxillary lattices with the prescribed scales in the lattice ensemble from Theorem 1. With high probability since $\hat{L}$ is good for coding semi norm-ergodic noise of power $\delta^{2}+\varepsilon$ [2] and applying [19] (Appendix V) to $\tilde{Y}$, ${\tilde{Y}}_{⊥}$ yields the result. □

**Proof.** (Theorem 1)

### Appendix B.1. Upper Bound for Singleton *S*

Take a nested lattice construction from [20] (Theorem 1), involving the following sets:

- *Coarse and fine encoding lattices*$L_{c},L_{1},\ldots ,L_{K}$ (base regions $B_{c},B_{1},\ldots ,B_{K}$) with each *k* has $L_{c}\subset L_{k}$ designed with nesting ratio $\frac{1}{n}\log\left|B_{c}\cap L_{k}\right|\to r_{k}$.
- *Discrete part auxiliary lattices*${\hat{L}}_{1},\ldots ,{\hat{L}}_{K}$ (base regions ${\hat{B}}_{1},\ldots ,{\hat{B}}_{K}$) with each ${\hat{L}}_{k}\subset L_{c}$ having nesting ratio $\frac{1}{n}\log\left|B_{c}\cap {\hat{L}}_{k}\right|\to \frac{1}{2}\log\delta_{k}^{2}$.
- *Initial residual part auxiliary lattice*${\hat{L}}_{0}^{\prime }$ (base region ${\hat{B}}_{0}^{\prime }$) with ${\hat{L}}_{0}^{\prime }\subset L_{K}$, nesting ratio $\frac{1}{n}\log\left|{\hat{B}}_{0}^{\prime }\cap L_{K}\right|\to \frac{1}{2}\log\sigma_{0}^{2}.$
- *Residual part auxiliary lattices*${\hat{L}}_{1}^{\prime },\ldots ,{\hat{L}}_{K}^{\prime }$ (base regions ${\hat{B}}_{1}^{\prime },\ldots ,{\hat{B}}_{K}^{\prime }$) with each ${\hat{L}}_{k}^{\prime }\subset L_{K}$, having nesting ratio $\frac{1}{n}\log\left|{\hat{B}}_{k}^{\prime }\cap L_{K}\right|\to \frac{1}{2}\log\sigma_{k}^{2}$.

The specified nesting ratios for the auxiliary lattices, $\sigma_{0}^{2},\sigma_{1}^{2},\ldots ,\sigma_{K}^{2},\delta_{1}^{2},\ldots ,\delta_{K}^{2}$ will be specified later.

*Initialization* 

Apply Lemma 1 to $U_{K}$, and label the resulting variables ${\tilde{Y}}_{0}:=\tilde{Y},\;{\tilde{Y}}_{0⊥}:={\tilde{Y}}_{⊥},\;\left({\hat{L}}_{0}^{\prime },{\hat{B}}_{0}^{\prime }\right):=\left({\hat{L}}^{\prime },{\hat{B}}^{\prime }\right),\;D_{0}:=n_{0}D_{},\;C_{0}:=C_{},\;\sigma_{0}^{2}:=\sigma^{2}.$ In addition, define $\delta_{0}^{2}:=n_{0}^{2},\;\beta_{0}=\frac{1}{n_{0}},\;{\hat{B}}_{0}:=n_{0}B_{c}$ Now,

$$U_{K}={\mathrm{mod}}_{B_{c}}\left(C_{0}+\beta_{0}{\tilde{Y}}_{0}+{\tilde{Y}}_{0⊥}\right)$$

so the support of $U_{K}$ is contained within:

$$\begin{array}{cc} S_{0}:= & \left[C_{0}+{\hat{B}}_{0}^{\prime }+\left(L_{c}/n_{0}+D_{0}\right)\right]\cap \left(B_{c}\cap L_{K}\right)\\ = & [C_{0}+{\hat{B}}_{0}^{\prime }+\beta_{0}\left[\left(D_{0}+L_{c}\right)\cap {\hat{B}}_{0}\right]\cap \left(B_{c}\cap L_{K}\right). \end{array}$$

*Support* *Reduction*

Iterate over steps s=1,…,K. For step *s*, condition on any event of the form ${\tilde{Y}}_{\left(s-1\right)}=\ell_{s}\in \left(D_{s-1}+L_{c}\right)\cap {\hat{B}}_{s-1}$, of which there are no more than $2^{n\cdot (\log\left(\delta_{s-1}^{2}\right)+\varepsilon )}$ choices due to the nesting ratio for ${\hat{L}}_{s-1}$ in $L_{c}$. Take $A_{s}:={STAGES}^{*}\;\left(\mathrm{var}\left({\vec{Y}}_{c}|{\left[{\vec{\alpha }}_{0},\ldots ,{\vec{\alpha }}_{s-1}\right]}^{†}{\vec{Y}}_{c}\right)\right).$ By [3] (Theorem 1), ${\vec{A}}_{s}:=A_{s}^{†}{\vec{Y}}_{c}$ is recoverable by processing $\left({\vec{A}}_{s-1},{\mathrm{mod}}_{n_{0}B_{c}}Y_{0}{\vec{U}}_{\left[K\right]},\vec{W}\right)$.

Now, apply Lemma 2 to $\left(Y,{\vec{\alpha }}_{},A\right)=\left({\tilde{Y}}_{(s-1)⊥},{\vec{\alpha }}_{s},A_{s}\right)$, and label the resulting variables ${\tilde{Y}}_{s}:=\tilde{Y},\;{\tilde{Y}}_{s⊥}:={\tilde{Y}}_{⊥},\;\left({\hat{L}}_{s},{\hat{B}}_{s}\right):=\left(\hat{L},\hat{B}\right),\;\left({\hat{L}}_{s}^{\prime },{\hat{B}}_{s}^{\prime }\right):=\left({\hat{L}}^{\prime },{\hat{B}}^{\prime }\right),\;D_{s}:=D_{},\;C_{s}:=C_{},\;\beta_{s}:=\beta ,\;\sigma_{s}^{2}:=\sigma^{2},\;\delta_{s}^{2}:=\delta^{2}.$ Now,

$$U_{K}={\mathrm{mod}}_{B_{c}}\left(\left[\sum_{t=0}^{s}C_{t}+\beta_{t}{\tilde{Y}}_{t}\right]+{\tilde{Y}}_{s⊥}\right)$$

so the support of $U_{K}$ is contained within:

$$S_{s}:=\left[\left[\sum_{t=0}^{s}C_{t}+\beta_{t}\left[\left(D_{t}+L_{c}\right)\cap {\hat{B}}_{t}\right]\right]+{\hat{B}}_{s}^{\prime }\right]\cap \left(B_{c}\cap L_{K}\right).$$

*Count Points* *in Estimated Supports*

By design, there are no more than $\prod_{t=0}^{s}2^{n\cdot (\frac{1}{2}\log\left(\delta_{t}^{2}\right)+\varepsilon )}$ possible choices for $S_{s}.$ Each $S_{s}$ has no more than $|{\hat{B}}_{s}^{\prime }\cap \left(B_{c}\cap L_{K}\right)|\leq 2^{n\cdot (r_{K}+\frac{1}{2}\log\left(\sigma_{s}^{2}\right)+\varepsilon )}$ points. Then,

$$H\left(U_{K}|{\vec{U}}_{\left[K-1\right]},\vec{W}\right)\leq \min_{s\in \{0\}\cup [K]}n\cdot \left(r_{s}+\frac{1}{2}\log\left(\sigma_{s}^{2}\right)+\sum_{t=0}^{s}\frac{1}{2}\log\left(\delta_{t}^{2}\right)+K\varepsilon \right).$$

*Bound* *Simultaneity*

An argument is given in Section B.1 for an upper bound on the singleton case. The argument uses a Zamir-good nested lattice construction with a finite amount of nesting criteria, and conditions on a finite amount of high-probability events. Then, the argument holds for all cases of this form simultaneously by using a Zamir-good nested lattice construction satisfying all of each case’s nesting criteria and conditioning on all of each case’s high-probability events.

The entropy for the general case $S=\left\{s_{1},\ldots ,s_{\left|S\right|}\right\},\;T=\left\{t_{1},\ldots ,t_{\left|T\right|}\right\}$ can be rewritten using the chain rule:

$$\begin{array}{cc} H\;\left({\vec{U}}_{S}|{\vec{U}}_{T},\vec{W}\right) & =\sum_{p=1}^{\left|S\right|}H\;\left({\vec{U}}_{s_{p}}|{\vec{U}}_{\{s_{m}:m<p\}\cup T},\vec{W}\right).□ \end{array}$$

## Appendix C. Sketch of Theorem 2 for Upper Bound on Entropy-Rates of Decentralized Processing Messages

**Proof.** *(Sketch)* Proceed identically as in the proof of Theorem 1 in Appendix B up until either Section B.1 *Initialization* if definition for $Y_{0}$ was changed, or repetition *s* where ${\vec{\alpha }}_{s}=\left[0,0,\ldots ,0,1\right]$ in Section B.1 *Support Reduction* if definition for ${\left({\vec{\alpha }}_{k}\right)}_{k}$ changed. In this portion, perform the following analysis instead. Compute:

(A1) $$\begin{array}{cc} D_{\left(msg\right)}:= & {\mathrm{mod}}_{B_{c,\mathrm{msg}}}\left({\left({\vec{a}}_{R}^{\left(msg\right)}+{\vec{a}}_{Z}^{\left(msg\right)}\right)}^{†}{\vec{Y}}_{\left[K-1\right]}-W_{\mathrm{msg}}\right)\\ = & {\mathrm{mod}}_{B_{c,\mathrm{msg}}}\left(\lambda^{\left(msg\right)}X_{\mathrm{msg}}^{n}+Y_{⊥}^{\left(msg\right)}-W_{\mathrm{msg}}\right)\\ = & {\mathrm{mod}}_{B_{c,\mathrm{msg}}}\left(\frac{\lambda^{\left(msg\right)}}{\gamma_{n}}{\mathrm{mod}}_{B_{c,\mathrm{msg}}}\left(M+W_{\mathrm{msg}}\right)+Y_{⊥}^{\left(msg\right)}-W_{\mathrm{msg}}\right)\\ = & {\mathrm{mod}}_{B_{c,\mathrm{msg}}}\left(\left(1+\frac{\lambda^{\left(msg\right)}}{\gamma_{n}}-1\right){\mathrm{mod}}_{B_{c,\mathrm{msg}}}\left(M+W_{\mathrm{msg}}\right)+Y_{⊥}^{\left(msg\right)}-W_{\mathrm{msg}}\right)\\ = & {\mathrm{mod}}_{B_{c,\mathrm{msg}}}\left(M+\left(\frac{\lambda^{\left(msg\right)}}{\gamma_{n}}-1\right){\mathrm{mod}}_{B_{c,\mathrm{msg}}}\left(M+W_{\mathrm{msg}}\right)+Y_{⊥}^{\left(msg\right)}\right). \end{array}$$

The additive terms in Equation (A1) are independent of one another, and the terms besides *M* have observed power $\delta_{\left(msg\right)}^{2}$. Choose the nesting ratio for $L_{f,\mathrm{msg}}$ in ${\hat{B}}_{s}$ as ${\hat{r}}_{s}:=\frac{1}{2}\log\left(\delta_{s}^{2}\right).$ Then, with high probability since ${\hat{L}}_{s}$ is good for coding semi norm-ergodic noise below power $\delta_{s}^{2}$ [2] and applying [19] (Appendix V) to the derivation in Equation (A1),

(A2) $$M\in L_{\left(msg\right)}:=\left(L_{f,\mathrm{msg}}\cap B_{c,\mathrm{msg}}\right)\cap {\mathrm{mod}}_{B_{c,\mathrm{msg}}}\left(D_{\left(msg\right)}+{\hat{B}}_{s}\right),$$

where $D_{\left(msg\right)}$ is computable by processing $\left({\vec{U}}_{\left[K-1\right]},\vec{W},{\left({\tilde{Y}}_{t}\right)}_{\left[s-1\right]}\right)$ and $\frac{1}{n}\log\left|L_{\left(msg\right)}\right|\leq {\hat{r}}_{s}+\varepsilon .$ Rearranging Equation (A2),

$$\begin{array}{cc} X_{\mathrm{msg}}^{n} & ={\mathrm{mod}}_{B_{c,\mathrm{msg}}}\left(M+W_{\mathrm{msg}}\right)\in L_{s}:={\mathrm{mod}}_{B_{c,\mathrm{msg}}}\left(L_{\left(\mathrm{msg}\right)}+W_{\mathrm{msg}}\right). \end{array}$$

Now, define:

$$\begin{array}{cc} {\tilde{Y}}_{s} & :=X_{\mathrm{msg}}^{n}, \end{array}$$

$$\begin{array}{cc} {\tilde{Y}}_{s⊥} & :=E_{⊥}\;\left({\tilde{Y}}_{(s-1)⊥}|X_{\mathrm{msg}}^{n}\right), \end{array}$$

$$\begin{array}{cc} C_{s} & :=E\;\left({\tilde{Y}}_{s-1}|{\vec{A}}_{s}\right), \end{array}$$

$$\begin{array}{cc} \beta_{s} & :=\frac{\mathrm{cov}\left({\tilde{Y}}_{(s-1)⊥},Y_{s}|{\vec{A}}_{s}\right)}{\delta_{s}^{2}}, \end{array}$$

$$\begin{array}{cc} \sigma_{s}^{2} & :=\mathrm{var}({\tilde{Y}}_{s⊥}). \end{array}$$

By construction, ${\tilde{Y}}_{s⊥}$ is the components in ${\tilde{Y}}_{(s-1)⊥}$ uncorrelated with $X_{\mathrm{msg}}^{n}$:

$$\begin{array}{cc} {\tilde{Y}}_{(s-1)⊥} & =\beta_{s}{\tilde{Y}}_{s}+{\tilde{Y}}_{s⊥}+C_{s}, \end{array}$$

$$\begin{array}{cc} {\tilde{Y}}_{s} & \in L_{s}. \end{array}$$

Proceed as in proof of Theorem 1. □

## Appendix D. Proof of Lemma 3 for Recombination of Decentralized Processing Lattice Modulos

**Proof.** By [3] (Theorem 1), a processing of ${\vec{U}}_{\left[K\right]}$ with high probability outputs

$$\begin{array}{c} a_{R}^{†}{\vec{Y}}_{\left[K\right]},\\ a_{R}\in \mathrm{image}{STAGES}^{*}\;\left(\Sigma \right)\subset R^{K}. \end{array}$$

One can assume the nested lattices for the message transmitter, $L_{f,\mathrm{msg}}⊃L_{c,\mathrm{msg}}$, are part of the lattice ensemble from Theorem 1, in particular ones finer than the main coarse lattice $L_{c}$ so that $L_{c}\subseteq L_{c,\mathrm{msg}}$ and:

$$\frac{1}{n}\log\left|L_{c,\mathrm{msg}}\cap B_{c}\right|\to {\hat{r}}_{c,\mathrm{msg}}\geq 0.$$

With this structure, then, for any $a_{Z}\in Z^{K}$, the encodings can be processed to produce (using lattice modulo’s distributive and subgroup properties)

$$\begin{array}{c} {\mathrm{mod}}_{L_{c,\mathrm{msg}}}\left({\mathrm{mod}}_{L_{c}}\left(a_{R}^{†}{\vec{Y}}_{\left[K\right]}+a_{Z}^{†}\left({\vec{U}}_{\left[K\right]}-{\vec{W}}_{\left[K\right]}\right)\right)\right)=\ldots \\ {\mathrm{mod}}_{L_{c,\mathrm{msg}}}\left({\mathrm{mod}}_{L_{c}}\left(a_{R}^{†}{\vec{Y}}_{\left[K\right]}+a_{Z}^{†}{\vec{Y}}_{\left[K\right]}\right)\right)=\ldots \\ {\mathrm{mod}}_{L_{c,\mathrm{msg}}}\left(a_{R}^{†}{\vec{Y}}_{\left[K\right]}+a_{Z}^{†}{\vec{Y}}_{\left[K\right]}\right)=\ldots \\ {\mathrm{mod}}_{L_{c,\mathrm{msg}}}\left(\lambda X_{\mathrm{msg}}+Y_{\mathrm{noise}}\right), \end{array}$$

where, in Equation (1), λ∈R and $Y_{\mathrm{noise}}$ is the conglomerate of noise terms independent of $X_{\mathrm{msg}}$ that are left over. For channels with additive Gaussian noise, $Y_{\mathrm{noise}}$ is a mixture of Gaussians and independent components uniform over good-for-quantization lattice base regions, so $Y_{\mathrm{noise}}$ will probably, for long enough blocklength, land inside the base of any coarse enough good-for-coding lattice [19] (Appendix V). □

## Appendix E. Proof of Corollary 1 for Achievability of the Decentralized Processing Rate

**Proof.** Fix any $r_{\mathrm{msg}},a_{Z},a_{R},\lambda ,\sigma_{\mathrm{noise}}^{2}$ from their definitions in Lemma 3 and any $\gamma^{2}\in \left(0,1\right]$. Choose a communications rate $R_{\mathrm{msg}}$ satisfying the criterion in the statement. Form an ensemble of lattices such as those described in Theorem 2, with nesting ratio for $L_{c}$ in $L_{\mathrm{msg}}$ as $\frac{1}{2}\log\left(1/\gamma^{2}\right)$ for γ∈(0,1) and $L_{\mathrm{msg}}=L_{c}$ if $\gamma^{2}=1$. This design means $\gamma_{n}^{2}:=\mathrm{var}{\mathrm{mod}}_{B_{c,\mathrm{msg}}}(X_{\mathrm{msg}}+W_{\mathrm{msg}})\to_{n}\gamma^{2}.$ Have the transmitter encode its message *M* into a modulation $X_{\mathrm{msg}}^{n}$ as described at the beginning of Section 3.1 using a dither $W_{\mathrm{msg}}$ of which all helpers and the decoder are informed. Have each *k*-th helper, k=1,…,K, process its observation vector into a lattice modulo encoding $U_{k}$ as described in Theorem 2 using a dither $W_{k}$ of which the decoder is informed. By Theorem 2, there exists a Slepian–Wolf binning scheme such that each *k*-th helper can process its message $U_{k}$ into a compression $U_{k}^{*}$ with $\frac{1}{n}H\left(U_{k}^{*}\right)<R_{k}$, and where a decoder can with high probability process the ensemble of compressions $\left(U_{1}^{*},\ldots ,U_{K}^{*}\right)$ along with dither side information $\left(\vec{W},W_{\mathrm{msg}}\right)$ into $\left(U_{1},\ldots ,U_{K}\right)$. Employ this binning scheme at each of the receivers, and have them each forward their compressions $U_{k}^{*}$ to the decoder. Have the decoder decompress $\left(U_{1}^{*},\ldots ,U_{K}^{*}\right)$ into $\left({\hat{U}}_{1},\ldots ,{\hat{U}}_{K}\right)$. By the previous statement, with high probability, $\left({\hat{U}}_{1},\ldots ,{\hat{U}}_{K}\right)=\vec{U}.$ Use the processing obtained from Lemma 3 on $\left({\hat{U}}_{1},\ldots ,{\hat{U}}_{K}\right)$, with high probability producing a signal:

$$U_{\mathrm{proc}}:={\mathrm{mod}}_{L_{c,\mathrm{msg}}}\left(\lambda X_{\mathrm{msg}}+Y_{\mathrm{noise}}\right).$$

*Decoding* Decoding proceeds similar to [4]. At the decoder, compute:

(A3) $$\begin{array}{cc} U_{\mathrm{proc}}^{\prime }:= & {\mathrm{mod}}_{L_{c,\mathrm{msg}}}\left(U_{\mathrm{proc}}-W_{\mathrm{msg}}\right)=\ldots \\ & {\mathrm{mod}}_{L_{c,\mathrm{msg}}}\left(\frac{\lambda }{\gamma_{n}}{\mathrm{mod}}_{L_{c,\mathrm{msg}}}\left(M+W_{\mathrm{msg}}\right)+Y_{\mathrm{noise}}-W_{\mathrm{msg}}\right)=\ldots \\ & {\mathrm{mod}}_{L_{c,\mathrm{msg}}}\left(\left(1+\frac{\lambda }{\gamma_{n}}-1\right){\mathrm{mod}}_{L_{c,\mathrm{msg}}}\left(M+W_{\mathrm{msg}}\right)+Y_{\mathrm{noise}}-W_{\mathrm{msg}}\right)=\ldots \\ & {\mathrm{mod}}_{L_{c,\mathrm{msg}}}\left(M+\left(\frac{\lambda }{\gamma_{n}}-1\right){\mathrm{mod}}_{L_{c,\mathrm{msg}}}\left(M+W_{\mathrm{msg}}\right)+Y_{\mathrm{noise}}\right). \end{array}$$

Recall that the fine codebook lattice $L_{f,\mathrm{msg}}$ has been designed to be good for coding and so that the coarse codebook lattice $L_{c,\mathrm{msg}}$ has a nesting ratio within it as $R_{\mathrm{msg}}$. This means that $L_{f,\mathrm{msg}}$ is good for coding semi norm-ergodic noise with power less than $\gamma_{n}^{2}2^{-2R_{\mathrm{msg}}}$. Notice $M⊥{\mathrm{mod}}_{L_{c,\mathrm{msg}}}\left(M+W_{\mathrm{msg}}\right)⊥Y_{\mathrm{noise}}$, where the first independence is by the crypto lemma [1]. This is to say that additive terms other than *M* in Equation (A3) are noise with power

(A4) $$\begin{array}{c} \mathrm{var}\left\{\left(\frac{\lambda }{\gamma_{n}}-1\right){\mathrm{mod}}_{L_{c,\mathrm{msg}}}\left(M+W_{\mathrm{msg}}\right)+Y_{\mathrm{noise}}\right\}=\ldots \\ \gamma_{n}^{2}\cdot {\left(1-\lambda /\gamma_{n}\right)}^{2}+\sigma_{\mathrm{noise}}^{2}. \end{array}$$

Furthermore, by [19] (Appendix V) on the noise, then it is probably in the base region of any lattice good for coding semi norm-ergodic noise with power less than Equation (A4). Then, ${\mathrm{round}}_{L_{f,\mathrm{msg}}}\left(U_{\mathrm{proc}}^{\prime }\right)=M$ with high probability if

$$\gamma_{n}^{2}\cdot {\left(1-\lambda /\gamma_{n}\right)}^{2}+\sigma_{\mathrm{noise}}^{2}<\gamma_{n}^{2}2^{-2R_{\mathrm{msg}}},$$

or, rearranging,

(A5) $$R_{\mathrm{msg}}<\frac{1}{2}\log\left\{\frac{\gamma_{n}^{2}}{{\left(\lambda -\gamma_{n}\right)}^{2}+\sigma_{\mathrm{noise}}^{2}}\right\}.$$

The limit of the right side of Equation (A5) equals Equation (3). □
